# Trends in exercise therapy research for neurological diseases: a bibliometric and visualization approach from 2000 to 2024

**DOI:** 10.3389/fneur.2024.1479731

**Published:** 2024-12-18

**Authors:** Jiacheng Zhang, Lielie Zhu

**Affiliations:** Department of Rehabilitation, Wenzhou TCM Hospital of Zhejiang Chinese Medical University, Wenzhou, China

**Keywords:** exercise therapy, neurological diseases, bibliometrics, visualization, CiteSpace, VOSviewer

## Abstract

**Background:**

Neurological disorders are one of the major global health burdens, and exercise therapy has been widely recognized as a beneficial intervention. However, the existing literature has primarily focused on summarizing the interventions, complications, and influencing factors, with relatively limited systematic comparisons and summaries.

**Methods:**

This study employed a bibliometric analysis approach, using VOSviewer and CiteSpace software to analyze the literature on the application of exercise therapy in neurological disorders from 2000 to 2024, including annual publication volumes, countries/regions, institutions, authors, journals, keyword co-occurrences, keyword clustering, keyword timelines, and keyword bursts.

**Results:**

The study found that the United States is the leading contributor in this field, and the University of Toronto and the University of Illinois are the most active research institutions. Keyword analysis revealed that the research hotspots in this field are concentrated on the role of exercise therapy in the prevention, treatment, rehabilitation, and improvement of quality of life for neurological disorders, and are gradually delving into its potential physiological mechanisms.

**Conclusion:**

This study provides valuable references for subsequent research in this field, helping to track the research frontiers and predict future research directions. Future research could further explore the specific mechanisms and clinical efficacy for different neurological diseases, providing more targeted evidence for clinical practice.

## 1 Introduction

The central nervous system (CNS), consisting of the brain and spinal cord, plays a crucial role in integrating the received information and coordinating various bodily functions (1). These neurological conditions encompass a wide range, including neurodevelopmental disorders, age-related neurodegenerative diseases, and emerging illnesses such as Parkinson’s disease, Alzheimer’s disease, multiple sclerosis, and post-COVID-19 cognitive impairment. According to a report published in The Lancet, an estimated 3.4 billion (95% UI 3.20–3.62) individuals worldwide were affected by neurological disorders in 2021, accounting for 43.1% (40.5–45.9) of the global population (2). Central nervous system diseases impose a significant health burden, resulting in mortality and disability (3). In 2021, neurological disorders were the leading cause of global disease burden, surpassing even cardiovascular diseases, and accounted for 443 million disability-adjusted life years (DALYs) lost due to disease, disability, and premature death (2). Additionally, factors such as stress, unhealthy lifestyles, work-rest imbalances, and environmental pollution can contribute to the development of neurological disorders across all age groups (4).

Physical activity (PA) has been widely recognized as a valuable intervention for various neurological conditions. Exercise therapy, a subcategory of PA, is a planned, structured, and repetitive activity with the ultimate or intermediate goal of improving or maintaining physical health (5). The 2018 review published in JAMA found that PA can promote healthy growth and development, enhance mood, cognitive function, and sleep quality, as well as lower the risk of various chronic diseases (6). A 2019 review found that exercise interventions have shown therapeutic benefits for a range of neurological disorders, including Parkinson’s disease, Huntington’s disease, and Alzheimer’s disease, regardless of the specific type and intensity of exercise (7). Furthermore, a 2021 meta-analysis revealed that exercise therapy has been shown to effectively improve aerobic capacity and muscle strength in the management of multiple sclerosis (8). Moreover, a 2020 review further underscored the diverse potential benefits of exercise for Alzheimer’s disease, including the prevention of associated risk factors (e.g., vascular dysfunction, obesity, diabetes) as well as the promotion of brain health, particularly through the muscle-brain axis (5). In recent years, there has been an increasing volume of research on the use of exercise therapy for the management of neurological disorders. However, the existing literature has mainly focused on summarizing the interventions, complications, and influencing factors, with relatively limited systematic comparisons and overviews of the current status and trends in this research area. Although this field has seen numerous review articles, traditional narrative reviews often rely on subjective assessments, lacking a quantitative approach. Such research methods that are based on personal experiences or biases can result in a less accurate and comprehensive evaluation of the current situation and trends (9).

Bibliometric analysis has become a widely adopted approach to identify research hotspots, analyze research outputs, and reveal trends. This is achieved by visualizing the internal connections among various pieces of information in the form of knowledge maps (10). Tools such as VOSviewer and CiteSpace have become essential for bibliometric studies. VOSviewer, co-developed by Professors Waltman and van Eck at Leiden University in the Netherlands, offers powerful visualization capabilities (11). On the other hand, CiteSpace is a web-based Java application created by Professor Chen’s team at Drexel University in the United States. It is specifically designed for data analysis and visualization, featuring a unique keyword burst detection function (12).

This study utilized VOSviewer and CiteSpace software to perform a bibliometric analysis of the literature on the application of exercise therapy in neurological disorders, covering the period from 2000 to 2024. The analysis examined various aspects, including annual publication volumes, countries/regions, institutions, authors, journals, keyword co-occurrences, keyword clustering, keyword timelines, and keyword bursts. This comprehensive analysis aimed to investigate the current status and research focal points in this field from both spatial and temporal perspectives. The findings are expected to help track the research frontiers and predict future research directions, thereby providing a valuable reference for subsequent studies in this domain.

## 2 Materials and methods

### 2.1 Searching strategy

The data analyzed in this study were extracted from the Web of Science Core Collection (WoSCC), a database published by Clarivate Analytics. To cover as many relevant articles as possible, we selected terms that are commonly used in the scientific literature to construct the search strategy. Terms linked to exercise therapy and neurological disorders were obtained from the Medical Subject Headings (MeSH) in the PubMed database for the purposes of this research. On May 19, 2024, a literature search was conducted, and the complete search strategy is presented in the Supplementary Table S1. The final dataset comprised 1,234 records, which were exported as plain text files, including information on publication year, title, author names, institutions, abstracts, keywords, and journal names.

### 2.2 Inclusion and exclusion criteria

Inclusion criteria: (1) the intervention population must be diagnosed with a neurological disorder; (2) research specifically addressed exercise therapy; (3) clinical studies on humans and should be published in English in the form of articles; (4) the article type was limited to original articles or reviews; (5) studies were published between January 1, 2000, and May 19, 2024.

Exclusion criteria: (1) the articles that did not pertain to exercise therapy in neurological disorders were excluded; (2) non-article formats, such as letters, were excluded; (3) articles written in languages other than English were excluded.

### 2.3 Data collection

The data curation and screening process for this study involved the following steps: (1) two reviewers from the team independently assessed the articles, removing those that did not align with the research focus. Any discrepancies were resolved through discussion. (2) The affiliations and country names were corrected and standardized to minimize the impact on the results. (3) The keywords were also standardized, as non-standardized keywords with variations in word forms, plurals, and singular versions could lead to the appearance of meaningless repetitions in the keyword co-occurrence analysis. For example, “Turkey” and “Türkiye” were unified as “Turkey,” “England” and “Scotland” were unified as “UK,” and “meta-analysis,” “metaanalysis,” and “Metaanalysis” were all standardized as “meta-analysis.”

### 2.4 Data analysis

The bibliometric data extracted from the database was stored in “download_*.txt” files for subsequent analysis. We employed Microsoft Excel to examine temporal trends in publication output. For the construction of visual network maps, we utilized VOSviewer version 1.6.18. This software, which implements a probabilistic data standardization method, enabled us to create comprehensive visualizations of publishing countries, contributing organizations, prominent authors, key journals and highly cited references. In these visualizations, node size correlates with connection degree, frequency of occurrence, and connection strength. The thickness of connecting lines represents the intensity of collaboration between nodes. Node colors denote distinct clusters within the network. This approach allowed for a multifaceted analysis of the research landscape in our field of study.

To construct knowledge mapping visualizations, we employed CiteSpaceV 6.3.R1 software. The analysis parameters were configured with time slicing set to 1, covering a temporal range from 2000 to 2024 at annual intervals. We utilized a time-based similarity algorithm to generate two key visualizations: timeline plots and keyword burst analysis. Timeline plots, presented as clustering diagrams, illustrate the historical evolution patterns of keyword-associated clusters, providing a chronological perspective on the field’s development. Keyword burst analysis serves as a crucial indicator for identifying emerging research trends, highlighting sudden increases in the usage frequency of specific terms. The integration of these two analytical approaches facilitated a comprehensive examination of shifts in research focal points over time and potential future trajectories within the field. This dual-method strategy allowed for a nuanced understanding of both the historical context and the emerging dynamics in our research domain.

## 3 Results

### 3.1 Analysis of annual publications

**Figure 1:**
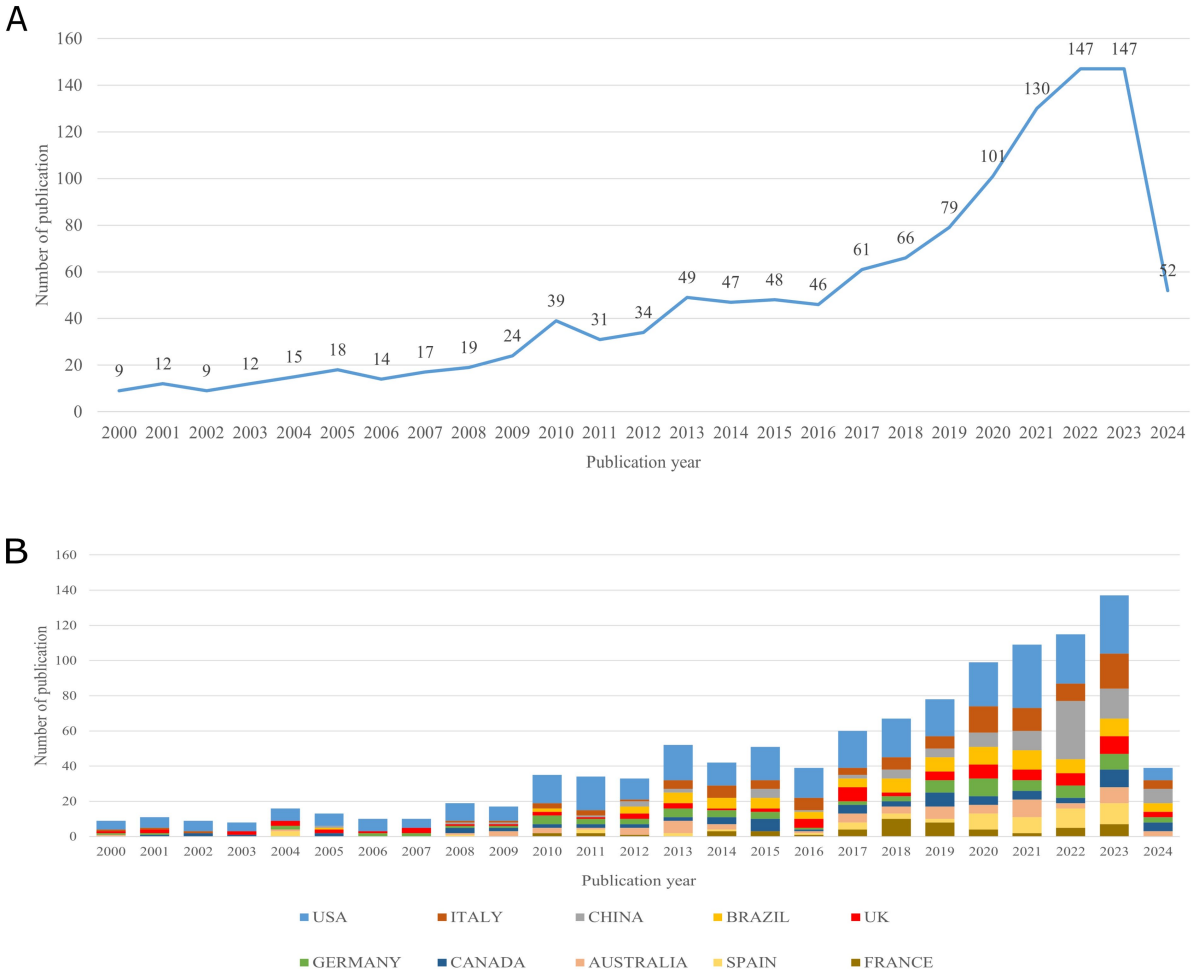
**(A)** Trends in the number of publications on exercise therapy in neurological disorders. **(B)** The growth trends of the top 10 productive countries on exercise therapy in neurological disorders.

As shown in Figure 1A, the publication output in the field of neurological disorders treated with physical therapy has been on an upward trend from 2000 to 2024. The annual publication volume can be roughly divided into three stages. During the period from 2000 to 2009, the annual publication count remained below 25 papers. From 2010 to 2016, the average annual publications increased to 42, indicating growing attention in this research area. After 2016, the publication output increased dramatically, suggesting a significant rise in the research focus on this topic.

### 3.2 Analysis of countries/regions

Based on the analysis using VOSviewer, a total of 66 countries/regions have published articles in this research field. Only the United States has published more than 150 papers. There are 39 countries/regions that have published more than 5 papers. Table 1 summarizes the publication output and betweenness centrality of the top 10 most productive countries/regions. The United States ranks first in terms of publication volume (376), followed by Italy (116), China (104), Brazil (94), and the United Kingdom (87). Betweenness centrality is used to describe the influence of a country in this field, and a value greater than 0.1 indicates significant impact. According to the betweenness centrality, the United States (0.31), Germany (0.19), and the United Kingdom (0.15) are considered to have a significant influence in this field, even though the publication volume of the UK and Germany are not among the highest. The top 5 countries/regions in terms of total citations are the United States (17,730), Canada (3,913), Italy (3,240), the United Kingdom (3,154), and Germany (2,953). The top 5 countries in terms of total link strength are the United States, the United Kingdom, Italy, Canada, and Germany.

**Table 1:** Top 10 countries/regions in number of papers published in exercise therapy in neurological disorders.

	Countries/regions	Documents	Centrality	Total citation	Total link strength
1	USA	376	0.21	17,979	198
2	Italy	116	0.12	3,294	105
3	People’s Republic of China	104	0.06	1,266	32
4	Brazil	94	0.06	1,170	37
5	UK	87	0.25	3,197	117
6	Germany	82	0.22	2,995	87
7	Canada	75	0.04	3,954	90
8	Australia	71	0.1	2,665	70
9	Spain	60	0.08	1,277	74
10	France	52	0.04	1,968	76

**Figure 2:**
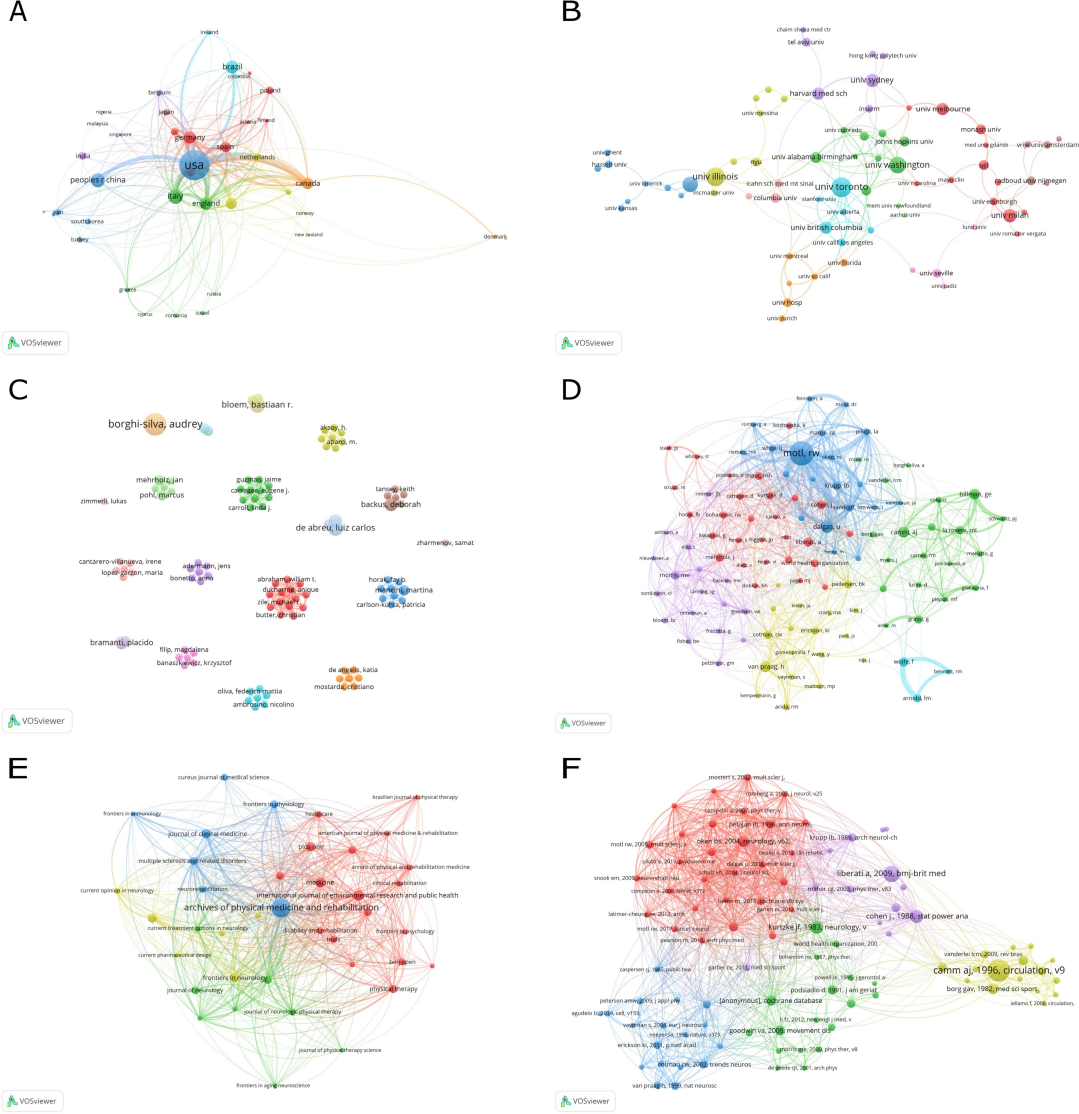
**(A)** Countries/regions cooperation analysis. **(B)** Institutions cooperation analysis. **(C)** Authors cooperation analysis. **(D)** Co-cited author analysis. **(E)** Journal analysis. **(F)** Co-references analysis. The node size indicates the number of publications; the color of the nodes indicates the clusters; the lines between the nodes indicate the existence of a collaborative relationship, and the thicker the line indicates the closer the relationship.

Figure 1B depicts the stacked bar chart showing the publication output growth trend of the top 10 most productive countries in this field from 2000 to 2024. The chart shows that the United States has the highest publication output, with a sustained upward trend. Italy and China are the next two most prolific, having begun focusing on this field around 2008. However, China’s growth has been particularly rapid, now approaching the levels of the United States and Italy. Figure 2A illustrates the collaborative landscape among nations and regions that have made substantial contributions to this research domain, specifically those with more than five publications. The visualization reveals eight distinct clusters among these 39 countries and regions, with interconnecting lines denoting co-authorship relationships. The varying thickness of these connections represents the intensity of collaboration, quantified by the total link strength (TLS) between each pair of countries or regions.

### 3.3 Analysis of institutions

Institutional collaboration network analysis using the VOSviewer software, we analyzed the institutional collaboration in the research field of applying movement therapy to neurological disorders. The analysis identified a total of 2,233 institutions involved in this research area. Figure 2B illustrates 72 of these institutions that have published at least 5 papers and collaborated with other institutions formed 11 distinct clusters. Table 2 presents the top 10 most prolific institutions in this research field. The list is led by the University of Toronto, followed by the University of Illinois, University of Washington, University of São Paulo, University of British Columbia, University of Milan, University of Sydney, University of Melbourne, Harvard Medical School, and the University of Alabama at Birmingham. This ranking reflects a global distribution of research excellence, spanning North America, South America, Europe, and Oceania.

**Table 2:** Top 10 institutions in number of papers published in exercise therapy in neurological disorders.

	Institution	Documents	Total citations	Total link strength	Country
1	University of Toronto	18	1,479	17	Canada
2	University of Illinois	17	833	8	America
3	University of Washington	15	1,399	10	America
4	University of São Paulo	14	218	5	Brazil
5	University of British Columbia	12	527	13	England
6	University of Milan	12	432	8	Italy
7	University of Sydney	12	364	9	Australia
8	University of Melbourne	11	514	5	Australia
9	Harvard Medical School	11	248	7	America
10	University of Alabama at Birmingham	10	230	10	America

Institutional research productivity and collaboration patterns according to the data presented in Table 2, the University of Toronto was the most prolific institution, contributing 18 publications, followed by the University of Illinois (17 publications) and the University of Washington (15 publications) in the United States. Overall, the top-ranking institutions were primarily from the United States, Australia, and Canada, indicating their leading roles in this research field. The analysis of institutional collaborations revealed that the University of Toronto, Columbia University, and the University of Washington had the strongest link strengths, suggesting a greater emphasis on inter-institutional collaborative research (Figure 2B).

### 3.4 Analysis of authors and co-cited authors

Our VOSviewer-based author analysis yielded significant insights into the field’s key contributors. Table 3 presents a comprehensive overview of the 15 most productive researchers and the 17 most cited scholars (with ties at the 17th position). The majority of high-output authors hail from the United States, Brazil, and various European nations, while the most cited researchers are predominantly based in the United States. Figure 2C depicts the collaborative networks among researchers with a minimum of three publications. Although large-scale collaborations were not evident, several smaller research clusters emerged. Notably, Motl, Robert W. stands out as the field’s leading contributor, boasting 10 publications and 616 citations, securing his position at the apex of both productivity and impact metrics. Figure 2D illustrates the interconnections among 107 researchers who have garnered over 20 citations each. Motl, Robert W. stands out with a clear lead over other highly cited authors, indicating his substantial contributions and influential role in this field.

**Table 3:** Top 15 authors in number of papers published and top 17 co-citied authors in exercise therapy in neurological disorders.

	Author	Documents	Total citations	Country		Co-cited author	Co-cited	Total link strength	Country
1	Motl, Robert W.	10	616	America	1	Motl, Robert W.	191	2,022	America
2	Borghi-Silva, A.	8	125	Brazil	2	Dalgas, Ulrik	77	1,001	Denmark
3	Bloem, Bastiaan R.	5	258	Netherlands	3	Van Praag, Henriette	65	345	America
4	De Abreu, Luiz C.	5	58	Brazil	4	Camm, Alan J.	64	250	England
5	Calabro, Rocco S.	5	34	Italy	5	Billman, George E.	62	408	America
6	Schlaich, Markus P.	4	265	Australia	6	Wolfe, Frederick	52	296	America
7	Dalgas, Ulrik	4	208	Denmark	7	Liberati, Alessandro	49	189	Italy
8	Langeskov-Christensen, M.	4	208	Danmark	8	Morris, Meg E.	47	364	Australia
9	Backus, Deborah	4	134	America	9	Krupp, Lauren B.	45	345	America
10	Feys, Peter	4	97	Belgium	10	La Rovere, Maria T.	45	320	Italy
11	Kool, Jan	4	67	Switzerland	11	Cohen, Jacob T.	44	227	Israel
12	Watts, Christopher R.	4	48	America	12	Arnold, Lesley M.	43	233	America
13	Raimundo, Rodrigo D.	4	42	Brazil	13	Thaut, Michael H.	43	141	Canada
14	Ploughman, Michelle	4	28	Canada	14	Cotman, Carl W.	41	310	America
15	Bramanti, Placido	4	12	Italy	15	Grassi, Guido	41	186	America
					16	Mehrholz, Jan	41	162	Germany
					17	Pedersen, Bente K.	41	395	Denmark

### 3.5 Analysis of journals

Our journal analysis, conducted using VOSviewer, identified 39 journals that have contributed significantly to this research field, each publishing a minimum of 5 articles. Table 4 highlights the top 10 most influential journals in this domain. Leading the list is *Archives of Physical Medicine and Rehabilitation*, which has made the most substantial contribution with 38 publications, accumulating an impressive 2,476 citations. Following closely is the *International Journal of Environmental Research and Public Health*, with 16 articles and 121 citations. These journals hold prominent positions in the field, being widely cited. The publication landscape of these 39 journals is visually represented in Figure 2E, revealing four distinct clusters. The journal landscape in this field exhibits a degree of clustering, with a few core journals being heavily cited, alongside more specialized, independent journals.

**Table 4:** Top 10 journals in number of papers published in exercise therapy in neurological disorders.

	Source	Documents	Total citations	Total link strength	IF (2023)
1	Archives of Physical Medicine and Rehabilitation	38	2,494	1,220	3.6/Q1
2	International Journal of Environmental Research and Public Health	16	123	344	4.6/Q2
3	Medicine	15	40	220	1.3/Q2
4	International Journal of Molecular Sciences	13	253	218	4.9/Q1
5	PLoS One	13	106	197	2.9/Q1
6	Journal of Clinical Medicine	13	105	277	3/Q1
7	Frontiers in Neurology	12	163	423	2.7/Q2
8	Cureus Journal of Medical Science	12	16	47	1/Q3
9	Cochrane Database of Systematic Reviews	11	1,018	599	8.8/Q1
10	Physical Therapy	11	368	246	3.5/Q1

IF, impact factor.

### 3.6 Analysis of highly cited and bursting references

**Figure 3:**
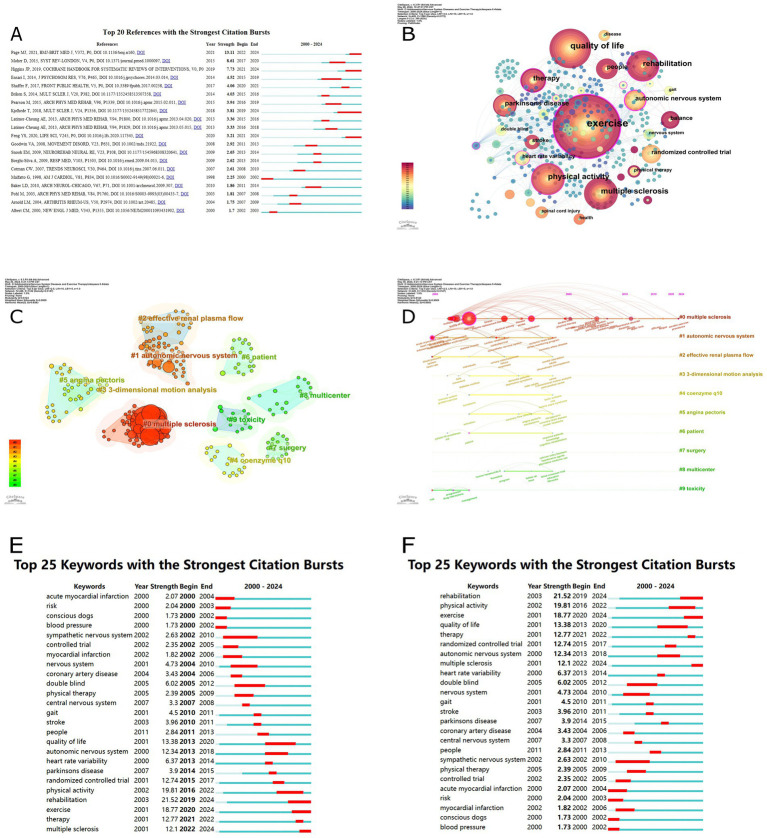
**(A)** Sorted by strengths of burst. **(B)** Keyword co-occurrence analysis. **(C)** Keyword clustering analysis. **(D)** Timeline of keyword clustering analysis. **(E)** Sorted by beginning year of burst. **(F)** Sorted by strengths of burst.

The bibliometric analysis revealed a total of 72,843 references, with 95 of them being cited more than 10 times. Table 5 presents the detailed information of the top 10 most cited references. The reference with the highest number of citations is “Heart rate variability—standards of measurement, physiological interpretation, and clinical use,” which has been cited 63 times. This is followed by “Preferred reporting items for systematic reviews and meta-analyses: the PRISMA statement” (43 citations) and “Statistical power analysis for the behavioral sciences” (33 citations). Figure 2F depicts the co-citation network of the 95 highly cited references. Furthermore, a reference burst analysis was conducted using CiteSpace, with the threshold parameter *Y* set to 0.5 and other parameters maintained at default values. This analysis identified 20 references exhibiting significant citation bursts, which are presented in Figure 3A. The visualization indicates that the highly cited articles are predominantly published within the past decade, suggesting an emerging influx of high-quality publications in this field in recent years.

**Table 5:** Top 10 co-references in number of papers published in exercise therapy in neurological disorders.

	Title	Year	Citations	Source/IF (2022)	First author	Type	Reference
1	Heart rate variability—standards of measurement, physiological interpretation, and clinical use	1996	63	Circulation/35.5/Q1	Camm, Alan J.	Guideline	(46)
2	Preferred reporting items for systematic reviews and meta-analyses: the PRISMA statement	2009	43	Annals of Internal Medicine/19.6/Q1 European Heart Journal/37.6/Q1	Moher, David	Guideline	(47)
3	Statistical power analysis for the behavioral sciences	1988	33		Cohen, Jacob	Book	(48)
4	Rating neurologic impairment in multiple sclerosis: an expanded disability status scale (EDSS)	1983	32	Neurology/7.7/Q1	Kurtzke, John F.	Article	(49)
5	Randomized controlled trial of yoga and exercise in multiple sclerosis	2004	26	Neurology/7.7/Q1	Oken, B. S.	Article	(50)
6	Psychophysical bases of perceived exertion	1982	25	Medicine & Science in Sports & Exercise/4.1/Q1	Borg, Gunnar A. V.	Symposium	(51)
7	The effectiveness of exercise interventions for people with Parkinson’s disease: a systematic review and meta-analysis	2008	23	Movement Disorders/7.4/Q1	Goodwin, Victoria A.	Review	(52)
8	Heart-rate recovery immediately after exercise as a predictor of mortality	1999	22	New England Journal Of Medicine/96.2/Q1	Cole, Christopher R.	Article	(53)
9	Effects of exercise training on fitness, mobility, fatigue, and health-related quality of life among adults with multiple sclerosis: a systematic review to inform guideline development	2013	21	Archives of Physical Medicine and Rehabilitation/3.6/Q1	Latimer-Cheung, Amy E.	Review	(54)
10	The timed “Up & Go”: a test of basic functional mobility for frail elderly persons	1991	21	Journal of the American Geriatrics Society/4.3/Q1	Podsiadlo, Danie	Article	(55)

IF, impact factor.

### 3.7 Analysis of keyword co-occurrence

The keyword co-occurrence analysis was conducted using CiteSpace, with the time slicing set to 1 and the top N keywords selected as 5. The resulting network included a total of 426 nodes and 1,332 links. Due to the density of the initial network, a pruning process was performed. Additionally, to address the issue of different author representations of the same keywords, node merging was carried out to consolidate synonymous terms. The resulting keyword co-occurrence network is presented in Figure 3B, where the purple outer ring indicates a betweenness centrality value greater than or equal to 0.1 for the corresponding node. Table 6 presents a comprehensive analysis of the 20 most significant keywords, ranked according to three key metrics: frequency of occurrence, betweenness centrality, and chronological order of initial appearance in the literature. The keywords with a betweenness centrality greater than or equal to 0.1 include “exercise” (0.36), “physical activity” (0.11), “rehabilitation” (0.1), “therapy” (0.13), “autonomic nervous system” (0.25), and “heart rate variability” (0.11). The main research types for exercise therapy in the neurological disorders are randomized controlled trials and double-blind studies. The primary disease areas investigated include multiple sclerosis, Parkinson’s disease, stroke, and spinal cord injury. The key research themes revolve around exercise, quality of life, physical activity, rehabilitation, therapy, autonomic nervous system, balance, heart rate variability, physical therapy, health, and gait.

**Table 6:** Keywords in top 20 research on exercise therapy for neurological disorders.

	Keywords	Frequency	Centrality	Year		Keywords	Frequency	Centrality	Year
1	exercise	379	0.36	2001	11	balance	27	0	2005
2	quality of life	155	0.09	2001	12	heart rate variability	21	0.11	2000
3	multiple sclerosis	102	0.04	2001	13	stroke	20	0.06	2003
4	physical activity	99	0.11	2002	14	physical therapy	14	0.01	2005
5	rehabilitation	87	0.1	2003	15	disease	12	0.02	2003
6	therapy	66	0.13	2001	16	spinal cord injury	12	0.09	2003
7	Parkinson disease	47	0.02	2007	17	double blind	11	0.05	2005
8	autonomic nervous system	39	0.25	2000	18	health	11	0.07	2003
9	people	37	0	2011	19	nervous system	11	0.02	2001
10	randomized controlled trial	33	0.06	2001	20	gait	8	0.06	2001

### 3.8 Analysis of keyword clustering

We employed CiteSpace software to perform keyword clustering analysis. The log-likelihood ratio (LLR) algorithm was utilized to categorize keywords into distinct thematic groups. The resulting network comprised 25 clusters, of which the top 10 clusters were selected for further analysis (Figure 3C). The modularity (*Q*) of 0.8124 and the weighted mean silhouette (*S*) of 0.9009 indicate that the keyword clustering modules derived from the data are statistically significant and highly reliable. The timeline analysis of the keyword clustering (Figure 3D) and the summary of the key terms associated with the top 10 clusters (Table 7) provide insights into the research themes and trends. Focusing on Cluster #0, which is related to “multiple sclerosis,” the silhouette value of 0.527 suggests a well-defined and reliable cluster. The main topics covered by Cluster #0 include multiple sclerosis, physical activity, myocardial infarction, autonomic nervous system, and quality of life.

**Table 7:** Top 10 keyword clusters.

Clusters	Size	Silhouette	Keywords
#0	54	0.527	multiple sclerosis; physical activity; myocardial infarction; autonomic nervous system; quality of life
#1	45	0.825	autonomic nervous system; myocardial infarction; heart rate variability; coronary artery disease; multiple sclerosis
#2	25	0.976	effective renal plasma flow; atrial natriuretic peptide; verapamil; norepinephrine; glomerular filtration rate
#3	23	0.994	3-dimensional motion analysis; joint position sense; nursing home residents; controlled clinical trial; functional reach
#4	19	0.98	coenzyme q10; encephalopathy; melas mitochondrial myopathy; strokelike episodes; mitochondrial encephalomyopathies
#5	19	0.942	angina pectoris; nitrates; cluster headache; glyceryl trinitrate; nitric oxide
#6	18	0.999	patient; incompetence; knowledge; acquisition; insufficiency
#7	17	1	surgery; platelet aggregation inhibitors; anesthetics; drug withdrawal; plants
#8	17	1	multicenter; blind crossover trial; education; placebo; amitriptyline
#9	15	0.902	toxicity; drug interactions; pentoxifyiline; peripheral diseases; pharmacokinetics

### 3.9 Analysis of keyword burst

A keyword burst analysis was conducted using CiteSpace, with the threshold parameter *Y* set to 0.5 and other parameters maintained at default values. This analysis identified 25 keywords exhibiting significant bursts. Figure 3E presents the data sorted by the beginning year of the research burst, while Figure 3F shows the data sorted by the strength of the research burst. From 2000 to 2011, keywords such as “double blind,” “nervous system,” “gait,” “stroke,” and “coronary artery disease” were commonly cited, with burst strengths reaching 6.02, 4.73, 4.5, 3.96, and 3.43, respectively. However, from 2012 to 2024, the focus shifted to “rehabilitation,” “physical activity,” “exercise,” “quality of life,” and “therapy,” with burst strengths reaching 21.52, 19.81, 18.77, 13.38, and 12.77, respectively (Figure 3E). Keywords associated with rehabilitation medicine and high-quality research methods have experienced a concentrated burst of citations in the past decade, indicating growing attention from leading experts and scholars in the rehabilitation field (Figure 3F).

## 4 Discussion

To our knowledge, this study presents the first comprehensive bibliometric analysis of research trends in exercise therapy for neurological disorders. A total of 1,224 publications from the WoSCC database, spanning the period from January 1, 2000 to May 19, 2024, were analyzed. The analyses, conducted using VOSviewer 1.6.18 and CiteSpace 6.3.R1, examined the temporal and spatial distribution, institutional and author contributions, and top journals in the field. Additionally, keyword analysis, cluster analysis, and burst hotspot analysis were used to identify current research hotspots and frontiers.

### 4.1 General description

The temporal analysis revealed a steady increase in publications over the years, with around 15 times more articles published in 2023 compared to 2000. The number of published studies remained consistently over 100 during the 2020–2023 period, suggesting a promising trend in this field, potentially driven by the high prevalence of neurological disorders. The relatively low publication output from 2000 to 2009 indicates that the study of exercise therapy for neurological conditions was in its early stages, followed by a gradual increase from 2010 to 2016, and a significant rise after 2017.

The United States was the leading contributor in this field, accounting for 33.76% of the total publications, which may be attributed to the early start of research in this area in the country (Table 1). The centrality values for the United States, Germany, and the United Kingdom were above 0.1, underscoring a significant emphasis on academic cooperation by researchers in these regions.

The United States led in the number of publications and total citations, emerging as the most collaborative country in the study of neurological disorders. This was attributed to a higher intensity of research compared to other nations. The Netherlands followed, ranking second in both the number of publications and levels of cooperation. Western countries and regions have exhibited strong cooperation, whereas the relatively low total link strength observed in China and Brazil indicates the need for further enhancement in research efforts. Furthermore, nearly all of the top 10 institutions are from countries or regions with the highest number of publications (Table 2), highlighting the robust academic capabilities of these nations in this field. Among the top 10 most prolific institutions, four were American.

Motl, Robert W., a professor at the University of Illinois Chicago, is the most prolific author in this field from the United States (Table 3). With an *H*-index of 75, he has made a substantial impact on the research community. Motl, Robert W. has been cited a total of 191 times and has a link strength of 2022, far exceeding other authors (Figure 2D). An analysis of Motl’s, Robert W. published papers reveals his focus on the diagnosis, treatment, rehabilitation, and quality of life for individuals with multiple sclerosis. He emphasizes that exercise can be an effective rehabilitation strategy to manage symptoms, restore function, enhance quality of life, promote overall wellness, and increase participation in daily activities (13). The most frequently cited article in this field is by Motl et al. (14). This study validated the Patient Determined Disease Steps (PDDS) as a reliable patient-reported outcome (PRO) measure of disability in multiple sclerosis (MS). The findings of this study have significantly influenced both clinical practice and subsequent research in the field.

The impact factor serves as a key metric for assessing journal quality. Among the top 10 most productive journals in this field, the Cochrane Database of Systematic Reviews stands out with the highest impact factor of 8.4. Its 11 published articles have garnered 1,018 citations, demonstrating the high quality and peer recognition of its publications (Table 4). Our analysis of the published literature reveals that this journal primarily focuses on high-quality systematic reviews, serving as a pivotal database for healthcare-related meta-analyses. It’s worth noting that journals across the impact factor spectrum play crucial roles in advancing exercise therapy for neurological disorders.

According to Table 5 and Figures 2F, 3A show that most of the highly cited articles were published 10 years ago, while most of the articles with high citation bursts were published in the last 10 years, indicating that this field is gradually attracting the attention of research scholars, and it may be a popular trend for future research.

### 4.2 Hot spots and frontiers

Keywords serve as the author’s distillation and generalization of the article’s content, reflecting its core focus. Analyzing keyword bursts can identify changes in research hotspots and emerging trends within a field. Multiple sclerosis (MS) has been a subject of intense focus and ongoing research among experts in the field (Figure 3D). Since 2015, the research hotspots have primarily focused on randomized controlled trials (15–18), physical therapy (19–22), rehabilitation (23, 24), exercise (7, 21), treatment (25, 26), and multiple sclerosis (27–29) (Figure 3E). These research areas have been widely studied and applied in both academia and clinical practice in recent years. Understanding these research hotspots can help develop targeted research plans and provide valuable references for academic exploration in the relevant fields.

An analysis of keywords over the past quarter-century reveals key research trends in exercise therapy for neurological conditions. One prominent focus has been the etiology of these disorders. For instance multiple sclerosis (MS) has been identified as an autoimmune condition characterized by central nervous system demyelination (30, 31). Regular exercise can exert anti-inflammatory effects in chronic inflammatory diseases such as MS by reducing pro-inflammatory cytokines and promoting anti-inflammatory cytokines thereby helping to modulate MS progression (32, 33). Parkinson disease (PD) is characterized by death of dopaminergic neurons in the substantia nigra. These include early onset of rapid eye movement sleep behavioral deficits and decreased sense of smell as well as late progression to the substantia nigra and other midbrain and basal forebrain structures (34). Parkinsonian symptom progression is directly proportional to autonomic dysfunction and postganglionic sympathetic damage is its main cause (35). According to a recent review type I muscle fibers are distinctly affected in MS and PD. In MS the impact is primarily on metabolic functions while in PD the effects are more pronounced in the structural characteristics of these fibers (36). (1) The evidence-based foundation and potential mechanisms of exercise therapy in neurological disorders. For patients with mild to moderate MS exercise therapy can increase aerobic capacity and muscle strength and improve the ability to perform activities of daily living while reducing fatigue and depressive states (37, 38). Exercise therapy has been shown to be beneficial in improving cognitive function and walking mobility among individuals with MS (39, 40). Aquatic therapy is considered an emerging therapeutic modality that can improve the quality of life in patients with MS (41). The long-term effects of exercise interventions on biological parameters (Irisin, BDNF, IL-6) are relatively modest in patients with MS (42). In Parkinson’s patients different forms of exercise therapy are beneficial for different movement aspects including gait and balance training aerobic exercise progressive resistance exercise treadmill exercise strength training tai chi and integrated exercise (7, 34). Research indicates that exercise therapy can mitigate damage to dopaminergic neurons in the midbrain and enhance basal ganglia function. This is achieved through adaptive changes in dopamine and glutamate neurotransmission. Consequently patients with early to mid-stage Parkinson’s disease may experience improved overall functionality and walking efficiency (37). High-intensity exercise therapy may prevent vascular dementia and Alzheimer’s disease but there is only modest evidence showing its significant impact on improving activities of daily living and cognition in people with dementia (37). Recent studies have shown that aerobic exercise improves cognitive and ADL and that combined exercise significantly improves strength fitness and balance functions in AD patients (7). This may be related to the fact that exercise therapy promotes an increase in the volume of the hippocampus as well as inducing anti-inflammatory effects (37). (2) Research on the effects of exercise therapy on the complications of neurological disorders. Exercise therapy has been demonstrated to be effective in improving depressive states and fatigue syndrome in patients with MS (43, 44). Interventions including gait and balance training as well as home-based or leisure exercise are effective in reducing fear of falling in individuals with PD and MS, respectively (45).

## 5 Conclusion

This study employed bibliometric analysis to comprehensively examine the current status and development trends of exercise therapy research in the field of neurological disorders. The results show that research in this field has been growing continuously, with countries such as the United States, Italy, and China leading in terms of paper output and academic influence. Keyword analysis indicates that the research hotspots in this field are concentrated on the role of exercise therapy in the prevention, treatment, rehabilitation, and improvement of quality of life for neurological disorders, and are gradually delving into its potential physiological mechanisms. This study provides valuable references for subsequent research in this field. In the future, this field may further focus on the application of exercise therapy in the personalized management and multidisciplinary collaboration of neurological disorders, providing more evidence for clinical practice.

## 6 Limitations

This study has the following limitations: (i) the study focused solely on the research trends of exercise therapy in the field of neurological disorders, without delving deeper into the specific mechanisms and clinical efficacy for different neurological diseases. Future research should further explore the underlying physiological mechanisms of exercise therapy to provide more targeted treatment strategies. (ii) This study only analyzed the published literature in the Web of Science database, which may have omitted relevant studies published in other databases or in non-English languages. A more comprehensive search strategy across multiple databases could provide a more complete picture of the research landscape. (iii) This study mainly adopted quantitative bibliometric analysis methods, lacking in-depth discussion of the research content and quality. Future research could combine qualitative research methods, such as systematic reviews and expert interviews, to present a more comprehensive understanding of the current status and development trends in this field, and provide more valuable references for clinical practice.

## Data Availability

The raw data supporting the conclusions of this article will be made available by the authors, without undue reservation.
