# Supplementary material for: Trends in exercise therapy research for neurological diseases: a bibliometric and visualization approach from 2000 to 2024
Source: Front Neurol. 2024 Dec 18;15:1479731. doi: 10.3389/fneur.2024.1479731 (PMC11688323; doi:10.3389/fneur.2024.1479731)
Supplement: Supplementary file 1 [file Table_1.docx]

**Supplementary Table**

**Table S1**

search strategy by 2024-05-19

| Serial numbers | Search modes | results |
| --- | --- | --- |
| #1 | TS=((Nervous System Diseases) OR (Disease, Nervous System) OR (Diseases, Nervous System) OR (Nervous System Disease) OR (Neurologic Disorders) OR (Disorder, Neurologic) OR (Disorders, Neurologic) OR (Neurologic Disorder) OR (Neurological Disorders) OR (Disorder, Neurological) OR (Disorders, Neurological) OR (Neurological Disorder) OR (Nervous System Disorders) OR (Disorder, Nervous System) OR (Disorders, Nervous System) OR (Nervous System Disorder)) | 254,504 |
| #2 | TS=( (Exercise Therapy) OR (Remedial Exercise) OR (Exercise, Remedial) OR (Exercises, Remedial) OR (Remedial Exercises) OR (Therapy, Exercise) OR (Exercise Therapies) OR (Therapies, Exercise) OR (Rehabilitation Exercise) OR (Exercise, Rehabilitation) OR (Exercises, Rehabilitation) OR (Rehabilitation Exercises)) | 95,912 |
| #3 | #1 AND #2 | 1,418 |
| #4 | #3 AND DOP=(2000-01-01/2024-05-19) AND LA=(English)) AND DT=(Article OR Review)) | 1,234 |
